# Pediatrics

**Published:** 1901-08

**Authors:** Maud J. Frye

**Affiliations:** Buffalo, N.Y.


### Pediatrics.

Conducted by MAUD J. FRYE. M. D., Buffalo, N. Y.

RARE CAUSES OF SUDDEN DEATH.

Metcalfe (*London Lancet*, May 25, 1901,) reports a case of sudden death from asphyxiation which the post mortem showed to have been caused by the blocking of the trachea by pieces of caseous gland. The patient, a girl of 13, had three years previously been treated for right-sided phthisis, but was considered cured. The right pleura was firmly adherent throughout. The trachea was blocked by pieces of caseous gland. On the right side, half an inch above the bifurcation, was a round lobe admitting a No. 12 catheter. This communicated with a sac containing a caseous gland similar to that found in the trachea. There was an old fibroid tuberculous lesion at the root of the right lung almost completely healed.

---

### ACUTE COLITIS IN CHILDREN.

CANTLEY (*London Lancet*, May 25, 1901,) reports an epidemic occurring in a certain London barracks. Six out of thirty children were attacked and three out of thirteen adult women. Four of the children died. Only one child under two years—an infant of three months—was attacked. In each case the onset was sudden, with vomiting, diarrhea and pyrexia. Vomiting was the most pronounced symptom. Anorexia was complete, the tongue sometimes clean, sometimes furred, with a dry, brown center. The stools were very frequent, small, green, offensive, and contained bright blood, mucus, and little or no fecal matter. There was no marked tenesmus. There was no tenderness along the colon. The abdomen was sometimes retracted and empty, sometimes moderately distended. There was considerable restlessness and nervous prostration, suggestive of a general toxemia. There was considerable fever throughout. Autopsies in two cases showed the inflammatory process was confined to the colon and the lower part of the ileum, the amount of inflammation in the ileum being of minor importance. In one case a bacteriological examination was made, the bacillus enteritidis sporogenes and the bacillus coli communis being isolated.

---

### TREATMENT OF PNEUMONIA IN CHILDREN.

FISCHER (*N. Y. Med. Jour.*, April 27, 1901,) gives his treatment of pneumonia in children as follows:

When fever begins give one drop doses of aconite, repeated every hour, with or without fresh spirit of mindercrus, of the latter half teaspoonful doses, until a general diaphoresis is produced. Give calomel till a liquid green stool is produced; it will not only exert a hydragogue effect, but will also stimulate the flow of bile and the action of the kidneys. This is extremely important in view of a possible complication of nephritis.

Remember that water will carry off toxic products through the gastro-intestinal tract and also stimulate the flow of urine. Give *ad lib.*

Give an occasional dose of castor oil to carry off the sputum, which has been swallowed.

If coal-tar antipyretics are given, guard them with musk or camphor. It is better to rely entirely on the cold pack, or if cerebral symptoms are marked, the tub bath will be found advantageous. In very young and delicate infants, it is advisable to give a few drops of Hoffman's anodyne immediately before giving the bath. Vigorous rubbing while the child is in the bath will stimulate the circulation and prevent collapse. A mustard foot-bath will invariably stimulate the circulation and promote diaphoresis. The duration of the bath should be about two minutes.

Intense dyspnea will be best relieved by dry cups. Ventilate thoroughly. If necessary to secure sleep codeine may be given, 1-10 grain, repeated in an hour or two, for a child of one year. For stimulants, when necessary, Tokay wine or good whiskey. Feed carefully, using milk, soups, with or without cereals, egg albumen.

---

### INFECTIOUS DISEASES AND THE MILK SUPPLY.

KOBER (*Am. Jour. of the Med. Sciences,* May, 1901,) presents conclusions drawn from the study of 330 outbreaks of infectious diseases traced to the milk supply. He points out some of the circumstances under which milk may be the cause of disease:

1. Sour milk, or milk which is on the point of turning is very liable in infants, children, or persons of feeble digestion to produce gastric and intestinal catarrhs of an acute or chronic character. In the more acute cases, in bottle-fed children, we have the phenomena of cholera infantum. The cause of untimely acidity of milk are improper feeding of the animals, dirty milk pans and rooms, unclean udders and teats, and a high temperature.

2. Milk may be rendered unfit for use and cause sickness in children by reason of improper food of the animal, or from use on the animal of strong remedial agents which may be excreted in the milk. The symptoms of poisoning from arsenic, copper, iodine, lead, mercury, tartar emetic, atropin, colchicum, croton oil, strychnin, veratrum viride, etc., have been thus observed.

3. Milk may be morbific as the product of a diseased animal. Inflammatory conditions of the udders and teats, especially the condition known as garget, are doubtless responsible for a large number of cases of pseudo-diphtheria and other septic infections. The milk of animals suffering from acute specific enteritis, puerperal and other septic fevers, foot and mouth disease, cow-pox, anthrax, pleuro-pneumonia, rabies and tetanus has also been known to produce sickness in the consumer.

4. Three out of twenty-five samples of Boston milk transmitted the germs of tuberculosis to the animals experimented on. The ordinary market milk of Copenhagen proved infectious in six out of twenty-eight rabbits.

5. Milk may acquire infectious properties after it leaves the cow. The writer has tabulated the histories of 330 outbreaks of infectious diseases spread through the milk supply; these outbreaks consist of 195 epidemics of typhoid fever, 99 epidemics of scarlet fever, and 36 epidemics of diphtheria.

Of the 195 epidemics of typhoid fever in 148 the disease prevailed on the farm. In 67 instances the infection probably reached the milk by percolation of the germs into the well-water with which the utensils were washed; in 16 of these cases there was intentional dilution. In 7 instances, the infection was attributed to the cows wading in sewage-polluted water and pastures; in 24 instances the dairy employees also acted as nurses; in 10 instances, the patients while suffering from a mild attack, or during the onset of the disease continued their work. Of the 99 epidemics of scarlet fever the disease prevailed in 68 instances, either at the dairy or milk farm. In 2 instances the infection was conveyed by means of infected bottles or milk cans left in scarlet-fever houses.

Of the 36 outbreaks of diphtheria tabulated, there is evidence that the disease prevailed at the dairy or farm in 13 instances. In 12 instances the disease is attributed directly to the cows having inflammatory conditions of the teats or udders. These instances may be regarded as typical examples of streptococcus or staphylococcus infection, giving rise to a form of follicular tonsillitis or pseudodiphtheria, often difficult to distinguish clinically from true diphtheria or scarlet fever.

---

### THE TREATMENT OF SUMMER DIARRHEA IN INFANTS.

KERLEY (*Medical News*, August 4, 1900,) writes about the practical points in the treatment of summer diarrhea. The two chief features of a rational treatment of summer complaint are elimination and diet. The former relieves the bacteria and their products from the digestive tract; the purpose of the diet is to give nourishment which will not furnish a favorable medium for the growth of the germs.

If no vomiting is present castor oil or calomel should be given, in order to clear the intestines. If the infant is vomiting this symptom must first be controlled by diet and lavage.

As regards diet, the first thing to be done is to stop the milk, no matter whether it is breast milk, cow's milk, or peptonised milk, or condensed milk, or any of the milk foods. The younger the patient the more imperative is the necessity of discontinuing the milk. In some cases it will be necessary to keep the milk away only for twenty-four hours, in others it has to be excluded for weeks. The diet in such cases should be as follows: if there is vomiting the stomach should be washed, and nothing whatever given for a few hours, when a teaspoonful of water may be tried. If the water is retained it may be given every fifteen minutes. The next step is to give increased quantities of dextrinised barley water at long intervals. If the case is one of diarrhea without vomiting he instructs the mother to give from 3 to 5 ounces of the dextrinised barley, either plain or salted, to which is added from 1 to 2 ounces of chicken, beef, or mutton broth.

The broths must be given cautiously, for in some patients they have a decided laxative effect. The writer does not use white of egg in water, because many children fail to digest it. The amount of food given may correspond to that given in health, but it should be given in smaller quantities at shorter intervals. Plain boiled water may be given at any time for thirst. The use of a proprietary preparation of diastase for the purpose of predigesting the barley is recommended. A too strong milk mixture must not be given on resuming the milk diet, and the latter should not be commenced until the stools are nearly normal. The increase of milk should be gradual. In cases in which a child cannot return to milk in the smallest quantity until October or November, scraped beef, beef juice, and predigested cereals are our main reliance. A teaspoonful or two of some soluble proprietary food may be added to each feeding of dextrinised barley. In breast-fed babies the attacks are usually milder, and they can often return to breast-milk after twenty-four or forty-eight hours.

The writer uses practically but four drugs in summer diarrhea, the castor oil and calomel, already mentioned, and bismuth subnitrate and opium. He uses large doses of bismuth subnitrate (12 to 20 grains) with aromatic syrup of rhubarb (3 minims) and water enough to make a drachm. Opium should not be given unless the passages are more than six or seven daily. In cases where there is much fever and prostration, 4-6 stools are of benefit as drainage, and opium and astringents should not be given. The best form of opium to give in such cases is Dover's powder, from $\frac{1}{4}$ to $\frac{1}{2}$ grain, every two or three hours for a child eight months of age. For the fever, packs, baths and sponging are all that is necessary. Alcohol disturbs the stomach, and strophantus, digitalis, or strychnin should therefore be given if stimulants are required. For uncontrollable vomiting a hypodermic injection of 1-100 gr. morphine with 1-600 gr. atropin may be given to a child one year old.

Irrigation of the colon, in the opinion of the writer, has been overdone. It is of great service when the stools are infrequent and foul. It is also useful in active cases with 6-8 passages, particularly if there is any blood or much mucus. The irrigations should be carried out at eight, twelve, or twenty-four hour intervals, depending upon the nature of the case. Boric acid or salt solution is to be used, or, if there is much mucus a solution of tannic acid, 1 per cent.

"It is not enough that the child be given sterilised milk and breast-milk; he must be made comfortable. The clothing should be of the lightest and on very hot days he should be in the open air in the shade, if in the country; if in the city, the coolest room in the house. Two or three fifteen minute spongings with water at 60° F. daily will make the child ever so much more comfortable. Further we know that the digestive capacity is lessened during the heated term and the milk should be reduced in strength from one-fourth to one-third on very trying days, adding water to replace the quantity removed. A child with summer diarrhea should not come in contact with other young members of the family, for summer diarrhea must unquestionably be placed in the list of communicable diseases."—*Abstracted in Pediatrics.*

